# *N*-(2,6-Di­chloro­phen­yl)-2-oxo-1,2-di­hydro­pyridine-3-carboxamide

**DOI:** 10.1107/S241431462300603X

**Published:** 2023-07-14

**Authors:** Ni Tu, Sihui Long

**Affiliations:** a School of Chemical Engineering and Pharmacy, Wuhan Institute of Technology, Wuhan, Hubei 430205, People’s Republic of China; Vienna University of Technology, Austria

**Keywords:** crystal structure, highly twisted conformation, lactam–lactam dimer, N—H⋯O hydrogen bonds

## Abstract

Crystals of the title compound, C_12_H_8_Cl_2_N_2_O_2_, were obtained by slow evaporation of an ethano­lic solution. An intra­molecular _amide_N—H⋯O=C_lactam_ hydrogen bond is observed. In the crystal, two mol­ecules pair up to form a centrosymmetric lactam–lactam dimers (LLD) by N—H⋯O=C hydrogen bonds, whereas the O=C_amide_ group of the mol­ecule does not participate in hydrogen bonding.

## Abstract 2

The title mol­ecule has a highly twisted conformation with the two aromatic rings being almost perpendicular to each other. Through mutual N—H⋯O hydrogen bonds, the mol­ecules form centrosymmetric lactam–lactam dimers in the crystal.

## Graphical abstract

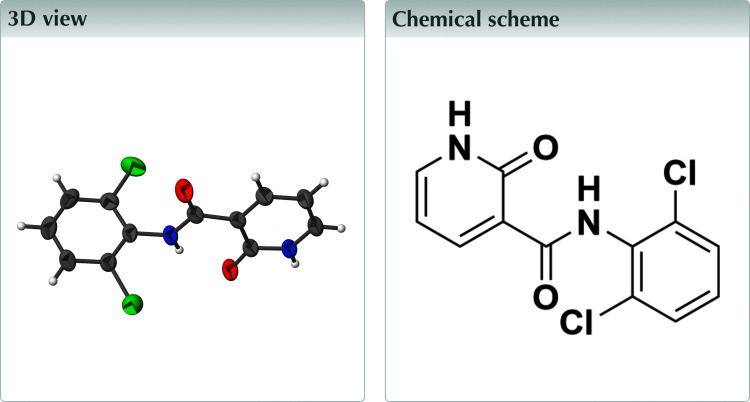

## Structure description

**Figure 1:**
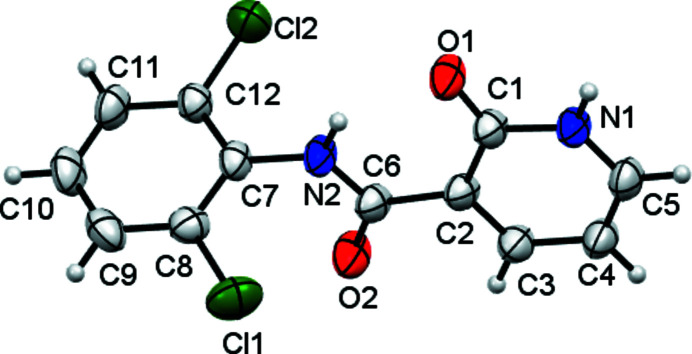
Mol­ecular structure of the title compound, with displacement ellipsoids drawn at the 50% probability level.

**Figure 2:**
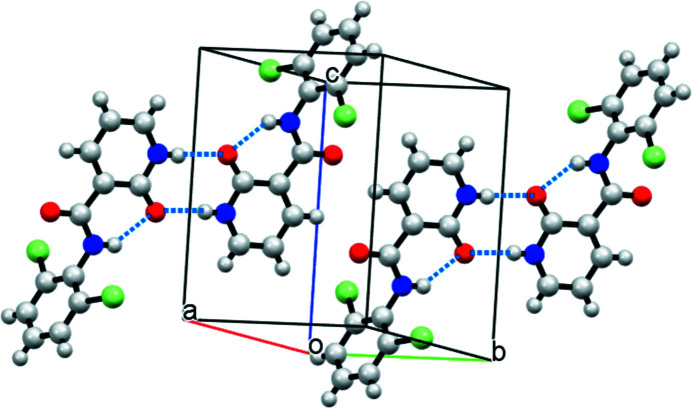
Packing of the mol­ecules in the crystal structure. N—H⋯O hydrogen bonds are indicated by dashed lines.

The mol­ecule of the title compound has two main functional groups, *i.e.* an amide (C6, N2, O2) and a lactam (C1, N1, O1) moiety (Fig. 1 ▸). An intra­molecular hydrogen bond is established between the amide NH group and the O atom of the lactam moiety (Fig. 2 ▸, Table 1 ▸). As a result of the large volume of the two chlorine substituents *ortho* to the C atom where the amide moiety is attached, the mol­ecule has a twisted conformation with a dihedral angle between the two aromatic rings of 70.68 (13)°.

In the crystal structure, at least two synthons, *i.e.* a lactam–lactam dimer (LLD) and a lactam–amide catemer, are possible. In two previous studies, both synthons were observed due to different substitution patterns on the mol­ecules (Liu *et al.*, 2020 ▸; Zhoujin *et al.*, 2021 ▸). In the crystal of the title compound, only the LLD synthon is observed in form of a centrosymmetric dimer established through _lactam_N—H⋯O=C_lactam_ hydrogen bonds, whereas the O=C_amide_ group of the mol­ecule does not participate in the formation of N—H⋯O hydrogen bonds (Table 1 ▸, Fig. 2 ▸).

## Synthesis and crystallization

**Figure 3:**
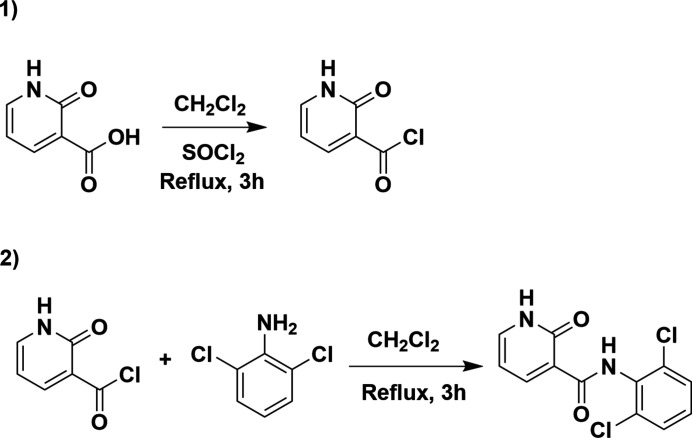
Synthesis scheme to obtain (**1**).

**Figure 4:**
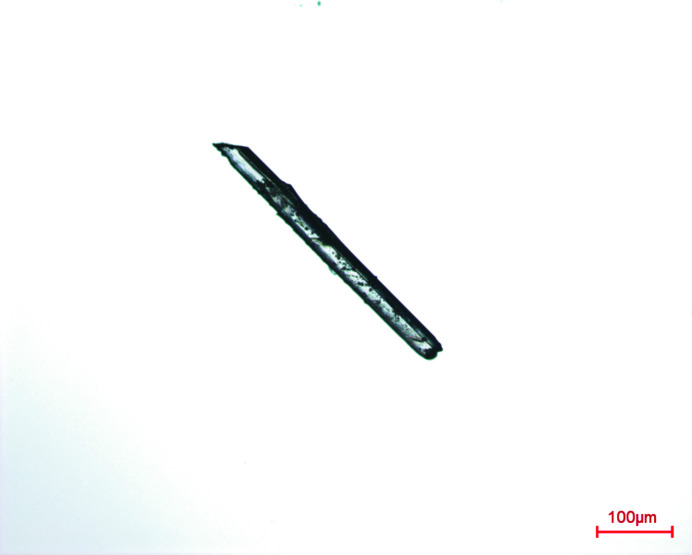
A representative crystal of (**1**).

The title compound was synthesized in two steps with 2-hy­droxy­nicotinic acid and 2,6-di­chloro­aniline as starting materials. First, 2-hy­droxy­nicotinic acid was converted into 2-hy­droxy­nicotinoyl chloride with thionyl chloride. Then 2-hy­droxy­nicotinoyl chloride was reacted with 2,6-di­chloro­aniline to provide the title compound (Fig. 3 ▸). Single crystals of the title compound were obtained through slow evaporation of a saturated ethano­lic solution. The details of the crystallization are as follows: about 30 mg of the compound was placed in a test tube, and an appropriate amount of solvent was added dropwise to dissolve the compound. The solution was filtered into a glass vial covered with a perforated parafilm (Hu *et al.*, 2018 ▸). Slow evaporation of the solution led to colorless single crystals in about a week (Fig. 4 ▸).

## Refinement

Crystal data, data collection and structure refinement details are summarized in Table 2 ▸. The H atom of the lactam moiety was refined freely.

## Supplementary Material

Crystal structure: contains datablock(s) global, I. DOI: 10.1107/S241431462300603X/wm4188sup1.cif

Structure factors: contains datablock(s) I. DOI: 10.1107/S241431462300603X/wm4188Isup2.hkl

Supporting information file. DOI: 10.1107/S241431462300603X/wm4188Isup3.cml

CCDC reference: 2280201

Additional supporting information:  crystallographic information; 3D view; checkCIF report

## Figures and Tables

**Table 1:** Hydrogen-bond geometry (Å, °)

*D*—H⋯*A*	*D*—H	H⋯*A*	*D*⋯*A*	*D*—H⋯*A*
N2—H2⋯O1	0.86	2.01	2.703 (3)	137
N1—H1⋯O1^i^	0.82 (4)	1.97 (4)	2.794 (3)	175 (3)

Symmetry code: (i) 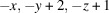 .

**Table 2:** Experimental details

Crystal data	
Chemical formula	C_12_H_8_Cl_2_N_2_O_2_
*M*_r_	283.10
Crystal system, space group	Triclinic, *P* 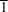
Temperature (K)	297
*a*, *b*, *c* (Å)	7.3730 (6), 8.0091 (6), 10.8545 (6)
α, β, γ (°)	97.296 (6), 95.228 (6), 102.149 (7)
*V* (Å^3^)	616.93 (8)
*Z*	2
Radiation type	Cu *K*α
μ (mm^−1^)	4.71
Crystal size (mm)	0.21 × 0.18 × 0.17

Data collection	
Diffractometer	XtaLAB Synergy R, DW system, HyPix
Absorption correction	Multi-scan (*CrysAlis PRO*; Rigaku OD, 2022 ▸)
*T*_min_, *T*_max_	0.140, 1.000
No. of measured, independent and observed [*I* > 2σ(*I*)] reflections	5370, 2135, 1943
*R*_int_	0.073
(sin θ/λ)_max_ (Å^−1^)	0.595

Refinement	
*R*[*F* ^2^ > 2σ(*F* ^2^)], *wR*(*F* ^2^), *S*	0.077, 0.210, 1.03
No. of reflections	2135
No. of parameters	168
H-atom treatment	H atoms treated by a mixture of independent and constrained refinement
Δρ_max_, Δρ_min_ (e Å^−3^)	0.74, −0.61

Computer programs: *CrysAlis PRO* (Rigaku OD, 2022 ▸), *SHELXT* (Sheldrick, 2015*a* ▸), *SHELXL* Sheldrick, 2015*b* ▸), *Mercury* (Macrae *et al.*, 2020 ▸) and *OLEX2* (Dolomanov *et al.*, 2009 ▸).

## full crystallographic data

### Crystal data

**Table:**

C_12_H_8_Cl_2_N_2_O_2_	*Z* = 2
*M**_r_* = 283.10	*F*(000) = 288
Triclinic, *P*1	*D*_x_ = 1.524 Mg m^−^^3^
*a* = 7.3730 (6) Å	Cu *K*α radiation, λ = 1.54184 Å
*b* = 8.0091 (6) Å	Cell parameters from 4779 reflections
*c* = 10.8545 (6) Å	θ = 4.1–76.2°
α = 97.296 (6)°	µ = 4.71 mm^−^^1^
β = 95.228 (6)°	*T* = 297 K
γ = 102.149 (7)°	Block, clear light colourless
*V* = 616.93 (8) Å^3^	0.21 × 0.18 × 0.17 mm

### Data collection

**Table:**

XtaLAB Synergy R, DW system, HyPix diffractometer	2135 independent reflections
Radiation source: Rotating-anode X-ray tube, Rigaku (Cu) X-ray Source	1943 reflections with *I* > 2σ(*I*)
Mirror monochromator	*R*_int_ = 0.073
Detector resolution: 10.0000 pixels mm^-1^	θ_max_ = 66.6°, θ_min_ = 4.1°
ω scans	*h* = −8→8
Absorption correction: multi-scan (CrysAlisPro; Rigaku OD, 2022)	*k* = −9→8
*T*_min_ = 0.140, *T*_max_ = 1.000	*l* = −12→12
5370 measured reflections	

### Refinement

**Table:**

Refinement on *F*^2^	Hydrogen site location: mixed
Least-squares matrix: full	H atoms treated by a mixture of independent and constrained refinement
*R*[*F*^2^ > 2σ(*F*^2^)] = 0.077	*w* = 1/[σ^2^(*F*_o_^2^) + (0.173*P*)^2^ + 0.0514*P*] where *P* = (*F*_o_^2^ + 2*F*_c_^2^)/3
*wR*(*F*^2^) = 0.210	(Δ/σ)_max_ < 0.001
*S* = 1.03	Δρ_max_ = 0.74 e Å^−^^3^
2135 reflections	Δρ_min_ = −0.61 e Å^−^^3^
168 parameters	Extinction correction: SHELXL-2018/3 (Sheldrick 2015b), Fc^*^=kFc[1+0.001xFc^2^λ^3^/sin(2θ)]^-1/4^
0 restraints	Extinction coefficient: 0.013 (4)
Primary atom site location: dual	

### Special details

**Table:**

Geometry. All esds (except the esd in the dihedral angle between two l.s. planes) are estimated using the full covariance matrix. The cell esds are taken into account individually in the estimation of esds in distances, angles and torsion angles; correlations between esds in cell parameters are only used when they are defined by crystal symmetry. An approximate (isotropic) treatment of cell esds is used for estimating esds involving l.s. planes.

### Fractional atomic coordinates and isotropic or equivalent isotropic displacement parameters (Å^2^)

**Table:**

	*x*	*y*	*z*	*U*_iso_*/*U*_eq_	
Cl1	0.09456 (12)	0.25172 (11)	0.21934 (9)	0.0689 (4)	
Cl2	0.33699 (11)	0.84435 (8)	0.03740 (7)	0.0606 (4)	
O1	0.0726 (3)	0.8647 (3)	0.38156 (19)	0.0573 (6)	
O2	0.4804 (3)	0.5753 (3)	0.32403 (18)	0.0601 (7)	
N1	0.2015 (3)	0.9462 (3)	0.5833 (2)	0.0451 (6)	
N2	0.2180 (3)	0.6370 (3)	0.2380 (2)	0.0449 (6)	
H2	0.128318	0.688602	0.249405	0.054*	
C1	0.1941 (3)	0.8550 (3)	0.4661 (2)	0.0416 (6)	
C2	0.3370 (3)	0.7567 (3)	0.4535 (2)	0.0394 (6)	
C3	0.4628 (4)	0.7595 (4)	0.5538 (3)	0.0458 (7)	
H3	0.553867	0.695703	0.544571	0.055*	
C4	0.4597 (4)	0.8558 (4)	0.6713 (3)	0.0498 (7)	
H4	0.546583	0.856661	0.739168	0.060*	
C5	0.3257 (4)	0.9469 (4)	0.6815 (3)	0.0478 (7)	
H5	0.319516	1.011107	0.758020	0.057*	
C6	0.3521 (4)	0.6494 (3)	0.3331 (2)	0.0414 (6)	
C7	0.2203 (3)	0.5413 (3)	0.1196 (2)	0.0390 (6)	
C8	0.1698 (3)	0.3609 (3)	0.0998 (3)	0.0441 (6)	
C9	0.1746 (4)	0.2670 (4)	−0.0165 (3)	0.0533 (8)	
H9	0.139775	0.146970	−0.028567	0.064*	
C10	0.2312 (4)	0.3524 (4)	−0.1133 (3)	0.0542 (8)	
H10	0.236139	0.289553	−0.190562	0.065*	
C11	0.2804 (4)	0.5292 (4)	−0.0971 (3)	0.0484 (7)	
H11	0.317890	0.586835	−0.162878	0.058*	
C12	0.2733 (3)	0.6204 (3)	0.0189 (2)	0.0405 (6)	
H1	0.118 (5)	0.999 (4)	0.589 (3)	0.047 (8)*	

### Atomic displacement parameters (Å^2^)

**Table:**

	*U*^11^	*U*^22^	*U*^33^	*U*^12^	*U*^13^	*U*^23^
Cl1	0.0707 (6)	0.0764 (6)	0.0693 (7)	0.0185 (4)	0.0188 (4)	0.0360 (5)
Cl2	0.0779 (6)	0.0474 (5)	0.0549 (6)	0.0110 (4)	0.0063 (4)	0.0078 (4)
O1	0.0613 (12)	0.0848 (15)	0.0359 (10)	0.0487 (11)	0.0014 (9)	−0.0050 (10)
O2	0.0628 (12)	0.0913 (15)	0.0394 (11)	0.0527 (11)	0.0088 (9)	−0.0018 (10)
N1	0.0521 (13)	0.0599 (13)	0.0319 (12)	0.0322 (10)	0.0106 (10)	0.0022 (10)
N2	0.0500 (12)	0.0643 (14)	0.0291 (12)	0.0346 (10)	0.0091 (9)	−0.0001 (10)
C1	0.0457 (13)	0.0540 (14)	0.0328 (13)	0.0253 (11)	0.0125 (11)	0.0055 (11)
C2	0.0446 (13)	0.0509 (14)	0.0308 (13)	0.0230 (10)	0.0130 (10)	0.0098 (11)
C3	0.0519 (14)	0.0557 (15)	0.0370 (14)	0.0278 (11)	0.0069 (11)	0.0063 (12)
C4	0.0600 (16)	0.0652 (17)	0.0310 (13)	0.0317 (13)	0.0034 (12)	0.0042 (12)
C5	0.0566 (15)	0.0610 (16)	0.0304 (13)	0.0234 (12)	0.0099 (11)	0.0032 (11)
C6	0.0472 (13)	0.0548 (14)	0.0311 (13)	0.0251 (10)	0.0142 (11)	0.0096 (11)
C7	0.0380 (12)	0.0509 (14)	0.0319 (13)	0.0213 (10)	0.0071 (10)	−0.0005 (11)
C8	0.0444 (13)	0.0512 (14)	0.0446 (15)	0.0223 (10)	0.0121 (11)	0.0119 (12)
C9	0.0537 (15)	0.0443 (13)	0.0626 (19)	0.0219 (11)	0.0054 (13)	−0.0062 (13)
C10	0.0576 (15)	0.0658 (17)	0.0430 (16)	0.0290 (13)	0.0135 (13)	−0.0082 (14)
C11	0.0531 (14)	0.0656 (17)	0.0315 (13)	0.0227 (12)	0.0139 (11)	0.0040 (12)
C12	0.0421 (12)	0.0461 (13)	0.0357 (13)	0.0158 (9)	0.0088 (10)	0.0029 (11)

### Geometric parameters (Å, º)

**Table:**

Cl1—C8	1.724 (3)	C3—C4	1.409 (4)
Cl2—C12	1.736 (3)	C4—H4	0.9300
O1—C1	1.244 (3)	C4—C5	1.350 (4)
O2—C6	1.223 (3)	C5—H5	0.9300
N1—C1	1.375 (4)	C7—C8	1.398 (4)
N1—C5	1.339 (4)	C7—C12	1.378 (4)
N1—H1	0.82 (4)	C8—C9	1.392 (4)
N2—H2	0.8600	C9—H9	0.9300
N2—C6	1.341 (3)	C9—C10	1.374 (5)
N2—C7	1.414 (3)	C10—H10	0.9300
C1—C2	1.448 (3)	C10—C11	1.369 (5)
C2—C3	1.359 (4)	C11—H11	0.9300
C2—C6	1.497 (3)	C11—C12	1.384 (4)
C3—H3	0.9300		
			
C1—N1—H1	113 (2)	O2—C6—N2	122.4 (2)
C5—N1—C1	125.1 (2)	O2—C6—C2	120.6 (2)
C5—N1—H1	122 (2)	N2—C6—C2	117.0 (2)
C6—N2—H2	119.2	C8—C7—N2	121.1 (2)
C6—N2—C7	121.6 (2)	C12—C7—N2	122.0 (2)
C7—N2—H2	119.2	C12—C7—C8	116.9 (2)
O1—C1—N1	119.5 (2)	C7—C8—Cl1	119.9 (2)
O1—C1—C2	126.0 (2)	C9—C8—Cl1	119.1 (2)
N1—C1—C2	114.5 (2)	C9—C8—C7	121.0 (3)
C1—C2—C6	122.5 (2)	C8—C9—H9	120.2
C3—C2—C1	119.7 (2)	C10—C9—C8	119.7 (3)
C3—C2—C6	117.8 (2)	C10—C9—H9	120.2
C2—C3—H3	119.0	C9—C10—H10	119.6
C2—C3—C4	122.1 (2)	C11—C10—C9	120.7 (3)
C4—C3—H3	119.0	C11—C10—H10	119.6
C3—C4—H4	121.3	C10—C11—H11	120.6
C5—C4—C3	117.5 (3)	C10—C11—C12	118.8 (3)
C5—C4—H4	121.3	C12—C11—H11	120.6
N1—C5—C4	121.1 (3)	C7—C12—Cl2	119.05 (19)
N1—C5—H5	119.4	C7—C12—C11	122.9 (2)
C4—C5—H5	119.4	C11—C12—Cl2	118.0 (2)
			
Cl1—C8—C9—C10	178.9 (2)	C5—N1—C1—O1	−179.7 (3)
O1—C1—C2—C3	179.2 (3)	C5—N1—C1—C2	−0.9 (4)
O1—C1—C2—C6	−1.2 (4)	C6—N2—C7—C8	76.0 (3)
N1—C1—C2—C3	0.5 (4)	C6—N2—C7—C12	−103.5 (3)
N1—C1—C2—C6	−179.9 (2)	C6—C2—C3—C4	−179.7 (2)
N2—C7—C8—Cl1	2.5 (3)	C7—N2—C6—O2	−2.1 (4)
N2—C7—C8—C9	−179.1 (2)	C7—N2—C6—C2	178.9 (2)
N2—C7—C12—Cl2	−0.6 (3)	C7—C8—C9—C10	0.5 (4)
N2—C7—C12—C11	178.6 (2)	C8—C7—C12—Cl2	179.79 (17)
C1—N1—C5—C4	0.9 (5)	C8—C7—C12—C11	−1.0 (4)
C1—C2—C3—C4	−0.1 (4)	C8—C9—C10—C11	−0.9 (5)
C1—C2—C6—O2	176.1 (3)	C9—C10—C11—C12	0.4 (4)
C1—C2—C6—N2	−4.8 (4)	C10—C11—C12—Cl2	179.8 (2)
C2—C3—C4—C5	0.0 (5)	C10—C11—C12—C7	0.5 (4)
C3—C2—C6—O2	−4.3 (4)	C12—C7—C8—Cl1	−177.95 (18)
C3—C2—C6—N2	174.8 (2)	C12—C7—C8—C9	0.4 (4)
C3—C4—C5—N1	−0.4 (5)		

### Hydrogen-bond geometry (Å, º)

**Table:**

*D*—H···*A*	*D*—H	H···*A*	*D*···*A*	*D*—H···*A*
N2—H2···O1	0.86	2.01	2.703 (3)	137
N1—H1···O1^i^	0.82 (4)	1.97 (4)	2.794 (3)	175 (3)

Symmetry code: (i) −*x*, −*y*+2, −*z*+1.
